# Dementia and Living Arrangements Among Mexicans and Mexican Americans

**DOI:** 10.1093/geroni/igab046.1843

**Published:** 2021-12-17

**Authors:** Phillip Cantu, Jiwon Kim, Sunshine Rote, Silvia Mejia-Arango, Mariana López-Ortega, Jacqueline Angel

**Affiliations:** 1 Sealy Center on Aging, University of Texas Medical Branch, Texas, United States; 2 UT Austin, Austin, Texas, United States; 3 University of Louisville, Louisville, Kentucky, United States; 4 Department of Population Studies El Colegio de la Frontera Norte, Tijuana, Baja California, Mexico; 5 National Institute of Geriatrics, National Institutes of Health, Mexico CIty, Distrito Federal, Mexico; 6 UT Austin, UT Austin, Texas, United States

## Abstract

This study compares the living arrangements of adults 85 and older in Mexican-origin adults in Mexico and the United States. The study uses 475 Mexican-Americans in five southwestern states (Hispanic Established Population for the Epidemiologic Studies of the Elderly, H-EPESE) and 1,710 Mexicans from 32 states in Mexico (Mexican Health and Aging Study, MHAS) to examine living alone vs living with others based on the presence of dementia controlling for demographic, health, and financial correlates. In both countries, more than 20% of respondents living alone have dementia. Dementia is associated with household extension in both countries. Homeownership increases household extension in Mexico but not the U.S. The findings show that individuals with dementia are more likely to live alone in the U.S. than in Mexico. Older individuals with dementia may be at elevated risk of isolation in later life if families or formal organizations cannot provide on-going assistance.

